# Short-Term Immobilization Promotes a Rapid Loss of Motor Evoked Potentials and Strength That Is Not Rescued by rTMS Treatment

**DOI:** 10.3389/fnhum.2021.640642

**Published:** 2021-04-26

**Authors:** Christopher J. Gaffney, Amber Drinkwater, Shalmali D. Joshi, Brandon O'Hanlon, Abbie Robinson, Kayle-Anne Sands, Kate Slade, Jason J. Braithwaite, Helen E. Nuttall

**Affiliations:** ^1^Lancaster Medical School, Health Innovation One, Lancaster University, Lancaster, United Kingdom; ^2^Department of Psychology, Faculty of Science & Technology, Lancaster University, Lancaster, United Kingdom

**Keywords:** rTMS, MEPs, immobilization, plasticity, muscle function

## Abstract

Short-term limb immobilization results in skeletal muscle decline, but the underlying mechanisms are incompletely understood. This study aimed to determine the neurophysiologic basis of immobilization-induced skeletal muscle decline, and whether repetitive Transcranial Magnetic Stimulation (rTMS) could prevent any decline. Twenty-four healthy young males (20 ± 0.5 years) underwent unilateral limb immobilization for 72 h. Subjects were randomized between daily rTMS (*n* = 12) using six 20 Hz pulse trains of 1.5 s duration with a 60 s inter-train-interval delivered at 90% resting Motor Threshold (rMT), or Sham rTMS (*n* = 12) throughout immobilization. Maximal grip strength, EMG activity, arm volume, and composition were determined at 0 and 72 h. Motor Evoked Potentials (MEPs) were determined daily throughout immobilization to index motor excitability. Immobilization induced a significant reduction in motor excitability across time (−30% at 72 h; *p* < 0.05). The rTMS intervention increased motor excitability at 0 h (+13%, *p* < 0.05). Despite daily rTMS treatment, there was still a significant reduction in motor excitability (−33% at 72 h, *p* < 0.05), loss in EMG activity (−23.5% at 72 h; *p* < 0.05), and a loss of maximal grip strength (−22%, *p* < 0.001) after immobilization. Interestingly, the increase in biceps (Sham vs. rTMS) (+0.8 vs. +0.1 mm, *p* < 0.01) and posterior forearm (+0.3 vs. +0.0 mm, *p* < 0.05) skinfold thickness with immobilization in Sham treatment was not observed following rTMS treatment. Reduced MEPs drive the loss of strength with immobilization. Repetitive Transcranial Magnetic Stimulation cannot prevent this loss of strength but further investigation and optimization of neuroplasticity protocols may have therapeutic benefit.

## Introduction

In the present paper, we investigated whether repetitive Transcranial Magnetic Stimulation (rTMS) to the primary motor cortex (M1) could attenuate the loss of motor excitability during limb immobilization. In clinical settings, immobilization of an injured upper or lower limb prevents the limb from moving during recovery from injury. A number of different immobilization methods can be used, including plaster casts, braces, or splints, which all hold joints or bones in place and are very effective at preventing muscle activation (Campbell et al., 2019). This prevention of muscle activation during immobilization promotes a rapid loss of muscle strength and mass, and it therefore represents an excellent model to study muscle atrophy (Gaffney et al., 2020).

Research on limb immobilization largely focuses on the effects within skeletal muscle itself (Wall et al., 2015, 2016; Crossland et al., 2019; Luo et al., 2020) but evidence indicates that the human brain is also affected (Langer et al., 2012). Indeed, neuromuscular function is governed by peripheral processes at skeletal muscle (Rudrappa et al., 2016) and central processes in the brain; specifically, the motor cortex (Campbell et al., 2019). It is known that limb immobilization causes a decrease in excitability of motor brain areas after as little as 8 h (Avanzino et al., 2011; Rosenkranz et al., 2014). This is a serious concern for individuals who are bed-bound, or older adults with reduced mobility. Upper limb immobilization promotes a decrease in the cortical thickness of the left M1 and somatosensory area and reduces fractional anisotropy of white matter tracts associated with the right hemisphere M1 (Langer et al., 2012). Immobilization also promotes a change in the interhemispheric balance of the homologous motor cortices toward increased control of the non-immobilized limb (Furlan et al., 2016). Collectively, these observations suggest a reorganization of the motor systems in the brain with immobilization (Langer et al., 2012).

Whilst some literature suggests that immobilization leads to decline in the peripheral motor pathways directly (Alves et al., 2013), there is no change or a decrease in resting membrane potential and no change in acetylcholinesterase activity in the neuromuscular junction after 4 weeks of immobilization (Booth, 1982). These observations suggest peripheral neuromuscular changes are not causative of muscle decline. Further, a recent systematic review (Campbell et al., 2019) suggests that muscle atrophy cannot fully explain the functional loss during immobilization and that central processes appear critical. Thus, the focus of this study was to target motor cortical activity to prevent immobilization-induced neurophysiologic decline.

After limb immobilization, reduced cortical excitability, and muscle strength can be rehabilitated through targeted physical training of the inactive body part (Clark et al., 2009; Brocca et al., 2015; Furlan et al., 2016). The loss of strength during immobilization can prolong clinical recovery and can impair physical function long-term (Gaffney et al., 2020). Loss of strength is associated with a greater risk of falls (Dhillon and Hasni, 2017) and bone fracture (Marty et al., 2017). If adults in later life experience bone fracture, the loss of strength might never be recovered, resulting in loss of muscle structure and function, and type II diabetes (Morley et al., 2014). It is crucial, therefore, that protective strategies are explored which mitigate against loss of cortical excitability and strength during limb immobilization.

Transcranial Magnetic Stimulation (TMS) is a non-invasive brain stimulation technique that uses a coil to apply brief magnetic pulses, which through the process of electromagnetic induction result in electrical currents in the brain that perturb neural activity. When repetitive pulses (rTMS) are applied to the motor cortex, it can result in long-term potentiation- or long-term depression-like effects, depending on whether the stimulation is high frequency (5 Hz or more) or low frequency (1 Hz), respectively (Ziemann, 2017). Such effects have been assumed to result from changes to Ca^2+^ influx through post-synaptic NMDA receptors that are induced by different stimulation frequencies (Huang et al., 2011). Repetitive Transcranial Magnetic Stimulation can produce significant clinical improvement in various neurological and psychiatric disorders, including but not limited to, post-stroke motor recovery, neuropathic pain, and depression (Rossini et al., 2015; Lefaucheur et al., 2020).

Indeed, high-frequency 20 Hz rTMS has been shown to confer significant increases in excitability in the motor pathway to the hand through increasing motor evoked potential (MEP) amplitudes in both healthy populations (Maeda et al., 2000a,b; Gangitano et al., 2002) and individuals with Parkinson's disease (Khedr et al., 2019). Motor excitability appears to be maximally enhanced following 20 Hz rTMS relative to lower frequency rTMS of 10 and 1 Hz (e.g., Jennum et al., 1995; Maeda et al., 2000a). Indeed, in subacute stroke patients, 20 Hz rTMS to M1 has also been linked to improvement in upper limb motor function (Kim et al., 2014), though for a review of rTMS and stroke see Fisicaro et al. (2019). Accordingly, these findings suggest that 20 Hz rTMS presents a useful candidate frequency for modulating M1 during limb immobilization.

In the present study, we investigated whether 20 Hz rTMS to M1 can facilitate cortical excitability and protect against skeletal muscle decline. We hypothesized that rapid declines in strength with immobilization are likely to be neural in their mechanism and are underpinned by a loss of excitability within the motor pathway to the hand, indexed as a reduction in magnitude of MEPs. Moreover, we hypothesized that by stimulating M1 using 20 Hz rTMS and thus artificially creating activity in the motor pathway, we could (centrally) attenuate the decline of motor excitability and decline of skeletal muscle, which would have significant implications for prehabilitation (Lambert et al., 2021) or rehabilitation.

## Methods

### Subjects

Twenty-four recreationally active young males gave written informed consent to participate in this study, which was approved by Faculty of Science and Technology Research Ethics Committee at Lancaster University (FST17065). Subjects were (mean ± SEM) 20.7 ± 0.5 year, 69.1 ± 1.8 kg body mass, and had a BMI of 22.1 ± 0.5 kg/m^2^. Anthropometric data are detailed in Table 1. All experimentation conformed to the seventh revision of the *Declaration of Helsinki (2013)*. The study was registered as a clinical trial on ClinicalTrials.gov, with the identifier: NCT04130581. Subjects were recruited on the Lancaster University campus and surrounding area, and testing took place at Lancaster University TMS Lab. The inclusion criteria were that subjects were healthy males (Rogers and Dhaher, 2017), aged 18–30 year, had a BMI of 19–25 kg/m^2^, were right-handed (Triggs et al., 1994), passed the Lancaster University TMS Safety Screening form (based on guidelines from Rossi et al., 2009), and could give written informed consent. Subjects were excluded if they presented with a recent history of musculoskeletal injury (<2 year of participation), or if they were taking any medication that could affect muscle metabolism or safety.

**Table 1:** Subject characteristics at baseline.

**Variable**	**Sham (*n* = 12)**	**rTMS (*n* = 12)**
Age (year)	20.8 ± 0.6	20.5 ± 0.8
Height (m)	1.77 ± 0.01	1.79 ± 0.03
Body mass (kg)	69.5 ± 2.7	70.5 ± 2.8
BMI (kg/m^2^)	22.2 ± 0.7	22.3 ± 0.9
Arm volume (L)	2.18 ± 0.38	2.10 ± 0.22

*Values denote mean ± SEM. All Sham vs. rTMS p > 0.05*.

**Figure 1:**
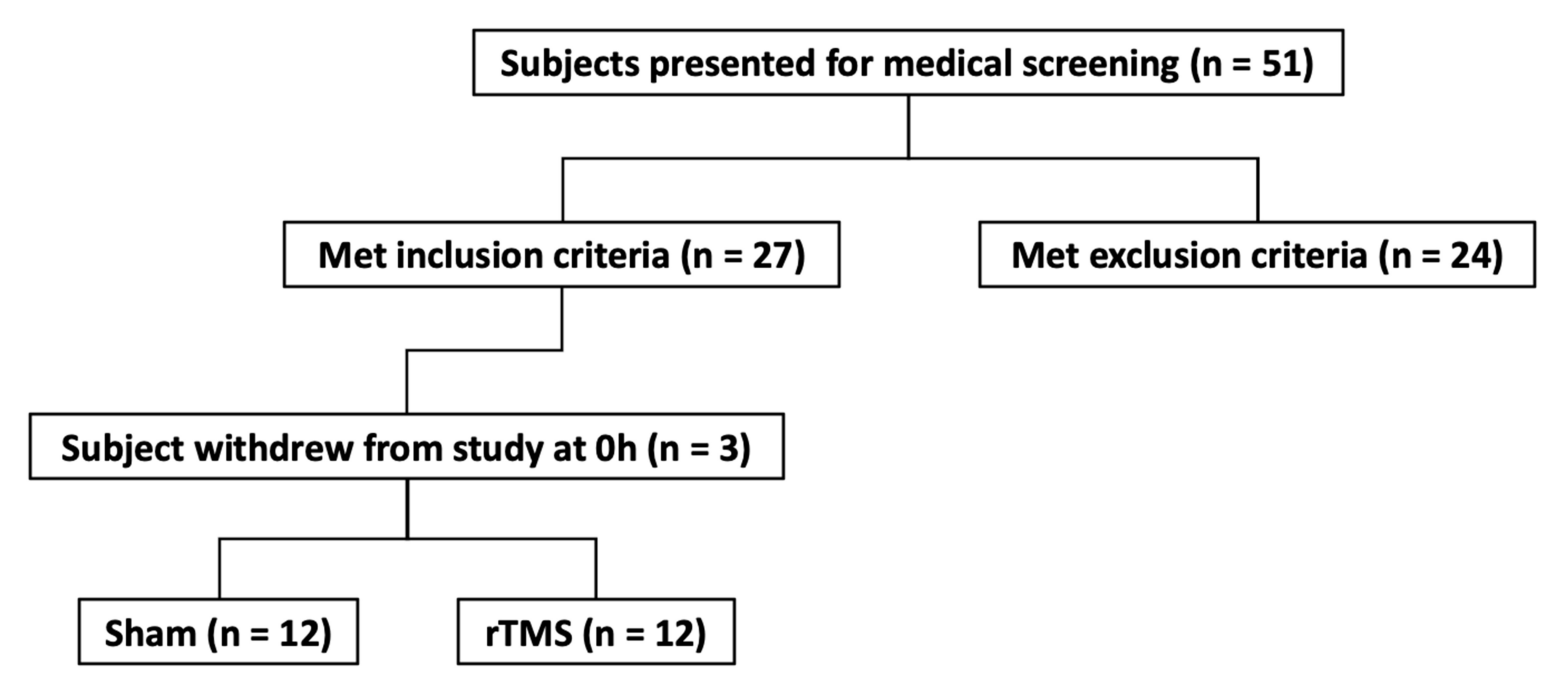
Flow chart of eligible participants and randomization to Sham or rTMS group.

Subjects were excluded from the rTMS intervention if they presented with a motor threshold that was not compatible with the upper safety limit of the intensity of the 20 Hz rTMS protocol. This was pre-determined by TMS safety guidelines (Rossi et al., 2009) i.e., if 90% of resting Motor Threshold (rMT) from First Dorsal Interosseous (FDI) exceeded 50% of maximum stimulator output (Figure 1). Based on the observed effect size of 0.8 associated with loss of cortical excitability with immobilization (Rosenkranz et al., 2014), an A-priori sample size calculation (power = 0.95; alpha = 0.05), indicated that the proposed study required a sample size of *n* = 9 per group. Thus, *n* = 12 per group were recruited to account for an anticipated 25% dropout rate during experimentation.

Subjects were randomized into two groups prior to collection of all baseline measurements at 0 h. Randomization was completed using a permuted block randomization design (block size of three), and a computer-based random number generator (Sealed Envelope, sealedenvelope.com, London, UK) for the first nine subjects. Stratified randomization was used for the final three subjects per group (Sham/rTMS) to ensure appropriate matching of groups.

### Experimental Design

The study sought to determine the loss of excitability in the motor pathway to the hand during 72 h unilateral limb immobilization, and its impact upon muscle strength and arm composition. A parallel design was applied (Sham vs. rTMS) with 72 h unilateral limb immobilization of the dominant arm using a shoulder sling and swathe, with the contralateral arm acting as a control (Triggs et al., 1994). The arm and hand were immobilized from the shoulder joint to the hand using a sling and swathe, which anchors the forearm to the torso and limits any activation of muscles in the arm (Magnus et al., 2010). Before and after the immobilization period, hand grip dynamometry (maximal grip strength), volume-displacement plethysmography, skinfold-calipers, and circumference measurements were performed to determine changes in strength and arm composition. Electromyography (EMG) of the FDI was completed during assessments of maximal grip strength. MEPs were determined from left and right FDI via single-pulse TMS at 0 h (before immobilization), and at 24, 48, and 72 h after immobilization.

**Figure 2:**
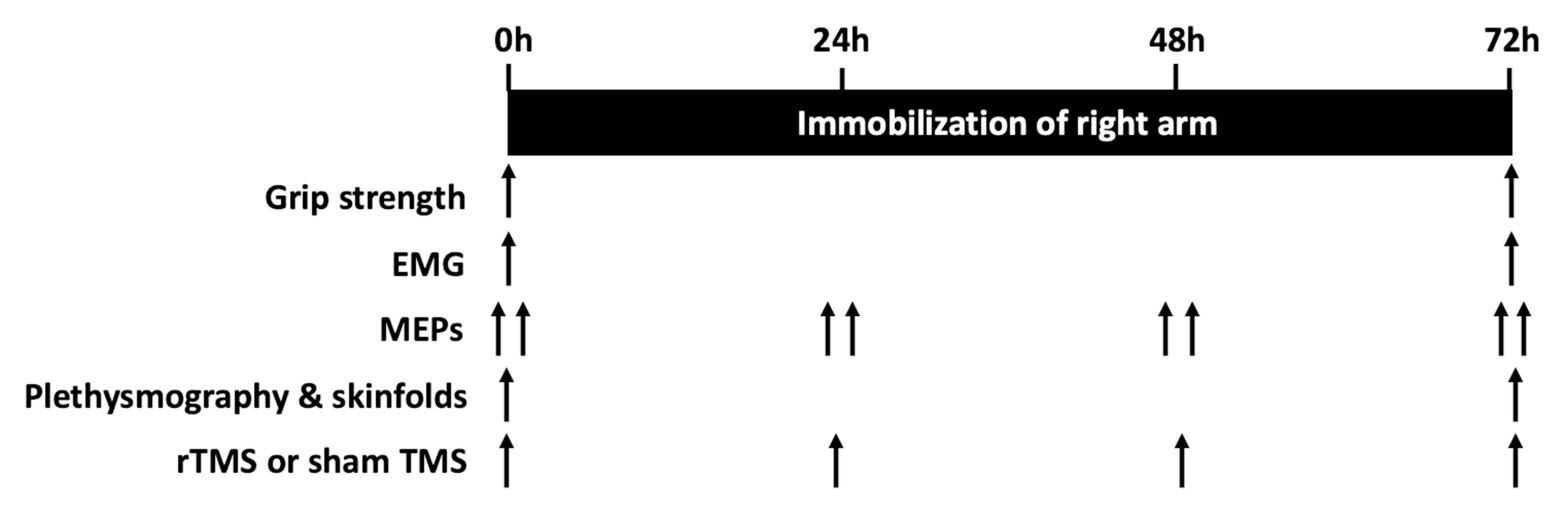
Schematic of the experimental design.

The rTMS group then received six × 1.5 s 30-pulse trains of 20 Hz biphasic rTMS with inter-train-intervals of 60 s via a 70 mm figure-of-eight coil attached to a DuoMAG XT-100 stimulator with Wasserman safety limits enabled (Deymed Diagnostic, Hradec Kralove, Czech Republic) to the hand area of left M1 before the sling and swathe were applied to immobilize the arm. This procedure was repeated daily at 24 and 48 h to promote cortical plasticity during immobilization (Lefaucheur et al., 2020). A further round of rTMS took place at 72 h after the sling removal and data collection. The Sham group received an identical rTMS protocol, but the coil was held 3–4 cm away from the head (Deng et al., 2013). All subjects were naïve to rTMS, and Sham or rTMS was delivered in a single-blind fashion. It was not necessary to unblind any participants during the trial. Maximal grip strength, arm volume, arm composition, and EMG activity during maximal grip strength testing were determined at baseline and following 72 h immobilization. Cortical excitability was evaluated using MEPs from the FDI elicited by single-pulse TMS, measured at 0, 24, 48, and 72 h. A schematic of the study design is detailed in Figure 2.

### Muscle Mass and Function

#### Maximal Strength and EMG Assessments

We assessed hand-grip strength using hand-grip dynamometry (Jamar hydraulic hand dynamometer, Jamar, Lafayette Instrument Company, USA) as previously described (Blomkvist et al., 2016), which has been shown to be highly responsive to forearm immobilization (Weibull et al., 2011).

#### Arm Composition

The composition of the upper arm and forearm were determined using both volume-displacement plethysmography (Brorson and Höijer, 2012) and skinfold calipers (Lohman et al., 1988) as previously described. In brief, to measure arm volume, the subject's arm was immersed up to their axilla with the volume of water displaced and weighed (Salter, Chadderton, UK) equal to the weight of the arm. At both baseline and after immobilization, the arm was inserted into the plethysmograph by the experimenter to limit any neural activation of the arm or hand muscles.

To confirm plethysmography measurements, measurements for forearm and upper arm circumference were also taken. The maximal circumference of the forearm was determined using a method previously described (Brorson and Höijer, 2012). The mid-point between the acromion process of the scapula and the olecranon process of the ulna was determined as the upper-arm mid-point, from which circumference was measured using a tape measure. To determine changes in subcutaneous fluid/fat, we measured skinfold sites on both the upper and lower arm using Harpenden Skinfold Calipers (Harpenden, Baty International, West Sussex, UK) before and after immobilization, as previously described (Lohman et al., 1988). In brief, skinfold measurements were taken at the biceps, triceps, anterior, and posterior forearm. All measurements for plethysmography and skinfolds were taken three times and a mean average was taken.

### Neurophysiologic Measures

#### Transcranial Magnetic Stimulation

For single-pulse stimulation to elicit MEPs, biphasic TMS pulses were generated by a DuoMAG XT-100 unit and delivered by a 70 mm diameter figure-of-eight coil. To ensure a posterior–anterior current flow using the biphasic stimulator, the coil was placed tangential to the skull at a 225-degree angle, which promotes posterior–anterior current flow in biphasic TMS stimulation relative to a 45-degree angle in monophasic stimulation (Sommer et al., 2006). Posterior–anterior current flow was required for effective MEP generation. Single-pulse and rTMS protocols were delivered using the same biphasic stimulator.

The hand area of each M1 was found by using the functional “hot spot” localization method (Möttönen et al., 2014). This location was then marked using Brainsight neuronavigation software (Rogue Research Inc., Montreal, Canada). For left M1, we also marked the 45-degree angle on the same hand location for use during rTMS for optimal efficacy of the plasticity protocol (Sommer et al., 2013). Localization of brain areas were performed at 0 h baseline, and the same co-ordinates were used for the visits at 24, 48, and 72 h.

In every session, the TMS procedures were identical. First, subjects received one block of single-pulse TMS to the hand area of left M1 (treatment hemisphere), and one block of single-pulse TMS to the hand area of right M1 (control hemisphere). The blocks of single-pulse TMS were presented at intensities that varied incrementally from 5 to 75% maximum stimulator output in 5% increments, with 2 stimuli applied per intensity (30 stimuli in total per block). There was a jittered inter-stimulation-interval of between 5.0 and 6.0 s. All MEPs were collected with the subject at rest, and muscle activation was visually monitored via EMG. The first TMS intensity interval at 0 h that elicited a robust MEP of 50 μV amplitude was taken as rMT. We used this adapted staircase procedure to efficiently estimate left M1 rMT at 0 h (Sen et al., 2017), and used the same procedure at each time point to track changes in excitability.

Second, rTMS or Sham was applied to the hand area of left M1 at a frequency of 20 Hz in six 1.5 s trains of 30 pulses, with inter-train-intervals of 60 s. The intensity of rTMS was delivered at 90% of rMT in right FDI (Sen et al., 2017), mean intensity = 41%, S.E.M. = 1.3%. In order to comply with safety guidelines, we did not apply the 20 Hz rTMS trains at intensities above 50% maximum stimulator output. This particular rTMS protocol and dosing procedure was selected based on evidence that it can increase motor excitability and generate effects that persist longer than lower frequency protocols (see section Introduction for details). The intensity and duration between pulse trains was informed by international safety guidelines (Rossi et al., 2009). The 90% threshold used for rTMS in the 0 h session was also used in the 24, 48, and 72 h sessions. Third, after the rTMS/Sham protocol, we repeated the 5–75% staircase procedure again, as described in the first step. MEPs were always collected from the left hemisphere followed by the right hemisphere both before and after rTMS. Single-pulse TMS blocks lasted for ≈3 min, and the rTMS/Sham block 5 min. All TMS was performed at rest, as we wanted to ensure that the muscles stayed inactive throughout the 72 h testing period, and testing under active contraction could theoretically have acted as an exercise countermeasure to loss of muscle strength. The same TMS coil was used throughout the experiment and for every session and every participant. All participants wore earplugs in both ears throughout all TMS/rTMS procedures (Tringali et al., 2012).

Electromyographic (EMG) activity was recorded from FDI using single-use, 30-mm diameter solid gel adhesive press-stud Kendall ECG electrodes with foam backs (Henleys Medical, Hertfordshire, UK) in a tendon-belly montage, with an electrode placed at the wrist serving as a common ground. The raw EMG signal was filtered between 1 and 2,000 Hz and sampled at 12,500 Hz online using a TruTrace 2-channel amplifier (Deymed Diagnostic, Czech Republic). Data epochs of 2 s were acquired and recorded using DuoMag rTMS software (version 6.2, Deymed Diagnostic, Czech Republic).

#### Statistics

To quantify excitability, MEP input-output functions were plotted offline in MATLAB (MATLAB R2016a, MathWorks, Massachusetts, USA) pre- and post-rTMS/Sham for each time-point. Area Under the Curve (AUC) was calculated for each input-output function as a measure of motor excitability. A larger AUC indicates greater motor excitability, and a smaller AUC indicates lower motor excitability. For the EMG acquired during maximal grip strength measurement, data were full-wave rectified offline in MATLAB and AUC was also computed. Mixed-model analysis of variance (ANOVA) was used to determine if there were statistically significant differences between all anthropometric measures, strength, EMG, and MEPs between groups and over time. When a significant main-effect was observed, *t*-tests were used to locate differences. Data were analyzed using GraphPad Prism (GraphPad Prism 8.0, GraphPad Software, Inc.). Data are presented as means ± SEM, and statistical significance was set at *p* < 0.05. In addition to *p*-values, Bayes Factor (BF) 10 values are reported. According to Jarosz and Wiley (2014), BF10 between 1 and 3 is regarded as weak evidence, 3–10 as moderate evidence, and >10 as strong evidence for the alternative hypothesis. Bayes factors were calculated using JASP v3.00.1. Reporting is aligned to CONSORT guidelines on the reporting of randomized controlled trials (http://www.consort-statement.org).

## Results

### The Effect of Immobilization on Motor Excitability

**Figure 3:**
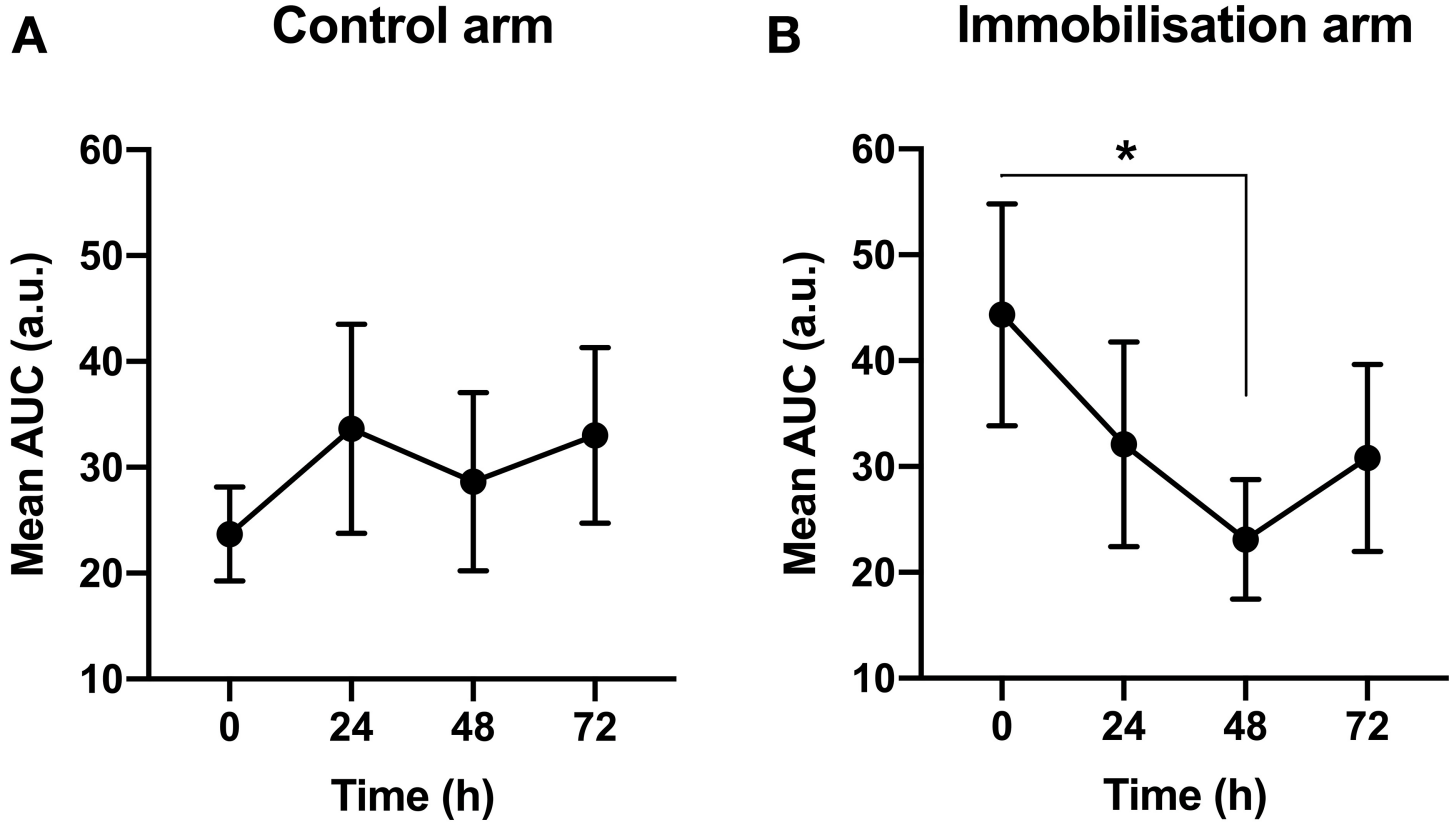
The effect of immobilization on motor excitabilit y. **(A)** In the control arm, there was no significant change in motor excitability across time. **(B)** In the immobilized arm, there was a reduction in motor excitability across time (*p* < 0.05). *0 vs. 48 h (−48%) (*p* < 0.05).

We first measured the change in MEPs in the Sham group to determine any effect of immobilization on motor excitability. Only MEPs collected before Sham treatment were used in the analysis to ensure data reflected a basal state. A repeated measures one-way ANOVA showed that in the control arm, there was no significant change in motor excitability across time [*F*_(2.09, 23.02)_ = 0.83, *p* > 0.05, BF_10_ < 1; Figure 3]. In the immobilized arm, however, there was a significant reduction in motor excitability across time [*F*_(1.88, 19.87)_ = 3.69, *p* < 0.05, BF_10_ = 2.75]. *Post-hoc* analysis revealed a significant difference between 48 and 0 h (−48% from 0 h; *p* < 0.05, BF_10_ = 4.65; Figure 3).

### The Effect of rTMS on Immobilization-Induced Loss of Motor Excitability

**Figure 4:**
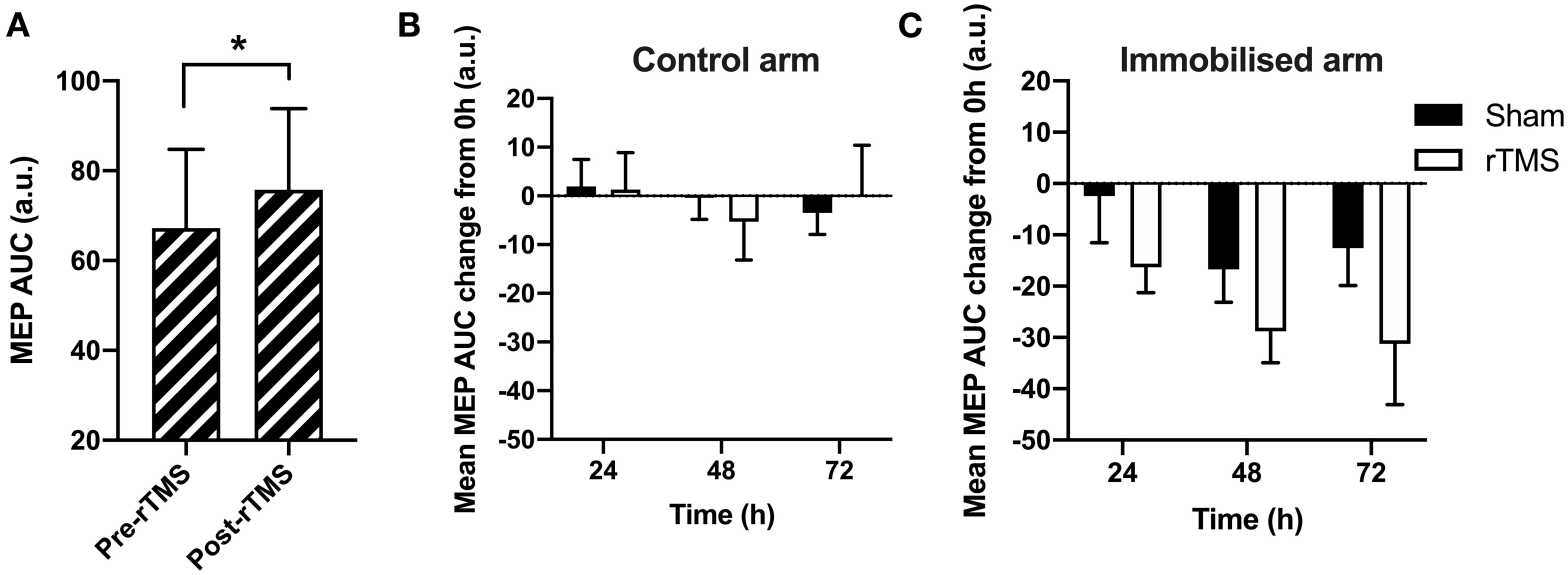
The effect of rTMS on the immobilization-induced loss of motor excitability. **(A)** There was a significant effect of rTMS on MEPs at 0 h (*p* < 0.05). **(B)** In the control arm, there was no significant change in MEP size across time or between Sham and rTMS (*p* > 0.05). **(C)** In the immobilized arm, there was a decrease in MEPs across time (*p* < 0.05), which did not differ between Sham and rTMS (*p* > 0.05). *p < 0.05.

We next sought to establish whether the rTMS protocol was efficacious in increasing motor excitability at 0 h baseline. A paired *t*-test indicated that there was a significant difference between motor excitability pre- vs. post-rTMS at 0 h (Figure 4A), indicating that the rTMS protocol was effective in increasing motor excitability [+13% from pre-rTMS; *t*_(11)_ = −1.77, *p* < 0.05, BF_10_ = 1.80].

To next determine whether the loss of motor excitability with 24, 48, and 72 h immobilization could be offset by rTMS, we compared the difference between MEPs collected during immobilization relative to MEPs at 0 h (baseline), across the rTMS and Sham groups. We sought to determine the decline in maximal excitability across immobilization and thus MEPs after rTMS are used hereafter. AUCs at each immobilization time-point were expressed relative to 0 h baseline. A negative score reflects a loss of motor excitability due to immobilization. This loss would be blunted in the immobilized arm if rTMS is effective in mitigating loss of motor excitability. In the control arm, there was no observable change in MEPs across time, or between Sham and rTMS groups (Figure 4B). A mixed-model ANOVA on control arm data with time and group as factors (Figure 4B) showed no effect for time: *F*_(2, 44)_ = 0.77, *p* > 0.05, BF_10_ < 1; group: *F*_(1, 22)_ < 0.01, *p* > 0.05, BF_10_ < 1; or interaction: *F*_(2, 44)_ = 0.65, *p* > 0.05, BF_10_ < 1. This lack of treatment effect and interaction indicates that rTMS had no change on the control arm data, as expected.

In the immobilized arm, Figure 4C shows how MEPs reduced across time, reflecting the immobilization effect seen in Figure 3B, whereby MEPs at 48 h were maximally reduced by immobilization relative to other time-points. A mixed-model ANOVA on data from the immobilized arm confirmed a significant main effect for time: *F*_(2, 44)_ = 3.57, *p* < 0.05, BF_10_ = 1.681. However, the effect of group was not significant: *F*_(1, 22)_ = 2.63, *p* > 0.05, BF_10_ < 1. The interaction between time and group was also not significant: *F*_(2, 44)_ = 0.19, *p* > 0.05, BF_10_ < 1. This lack of group effect and interaction between group and time on the immobilized arm indicates that rTMS did not significantly change the excitability in the immobilized motor pathway, contra to our prediction.

### The Effect of rTMS on the Loss of EMG Activity During Immobilization

**Figure 5:**
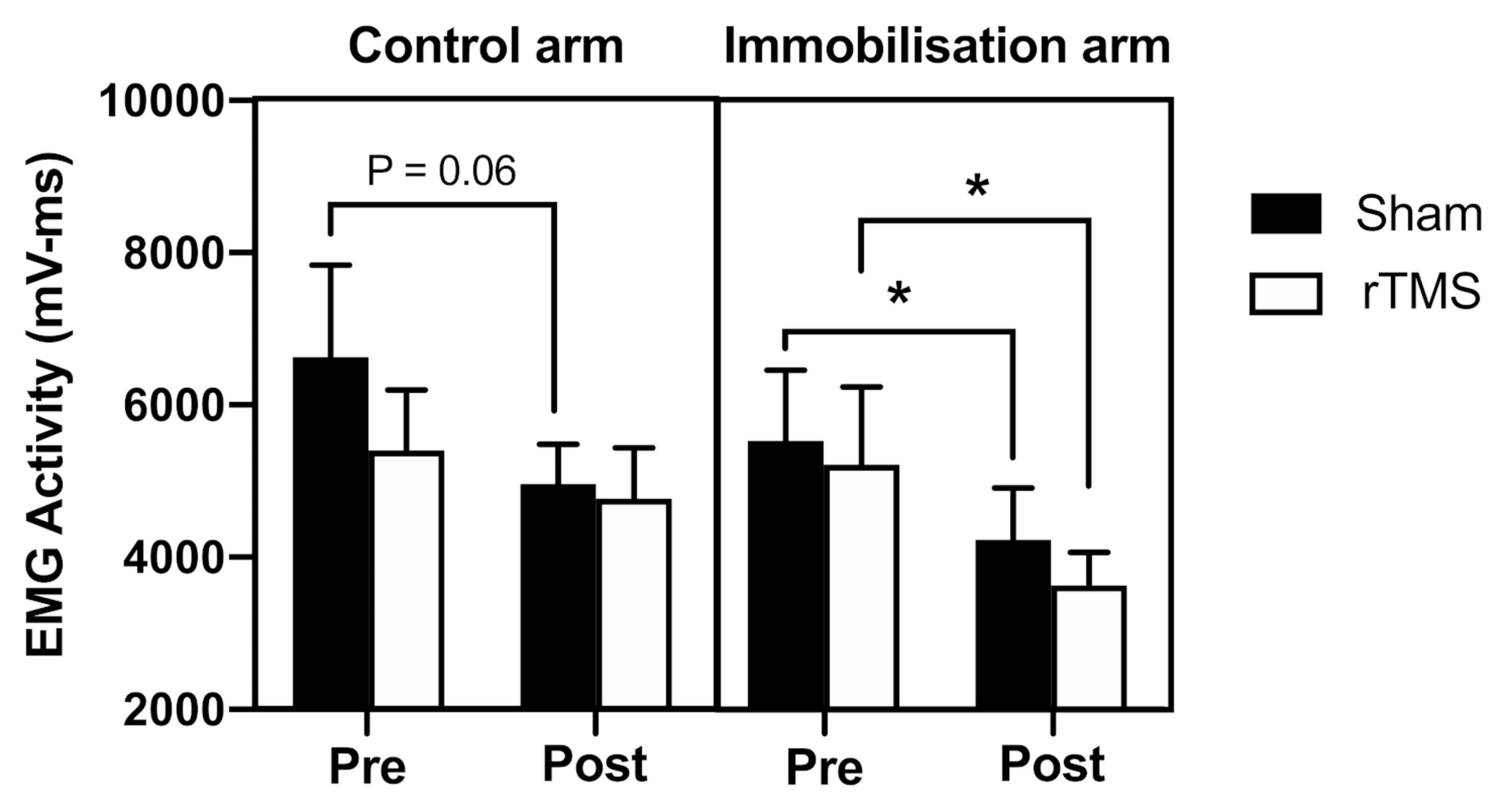
Effect of rTMS on immobilization-induced loss of EMG activity. In the control arm, EMG activity was reduced in the Sham group across the 72 h (trend; *p* = 0.06) but not in the rTMS group (*p* > 0.05). In the immobilized arm, there was a loss of EMG activity in the Sham group (*p* < 0.05) and this was not prevented with rTMS (*p* < 0.05). *p < 0.05.

Figure 5 shows the changes in EMG activity during immobilization. A mixed-model ANOVA with time (pre vs. post) and group (rTMS control arm; rTMS immobilized arm; Sham control arm; Sham immobilized arm) confirmed a significant reduction in EMG activity over time [*F*_(1, 44)_ = 7.57, *p* < 0.01, BF_10_ = 6.57]; however, there was no significant difference between rTMS and Sham groups [*F*_(3, 44)_ = 0.73, *p* > 0.05, BF_10_ < 1; Figure 5]. In the control arm receiving Sham, there was evidence of a reduction in EMG activity across the 72 h immobilization (−25%, *p* = 0.06, BF_10_ = 1.61). Interestingly, in the control arm of the group that received rTMS, there was no evidence of a reduction in EMG activity (−12%, *p* > 0.05, BF_10_ < 1). In the immobilized arm that received Sham, there was a similar reduction in EMG activity to the control arm (−24%, *p* < 0.05, BF_10_ = 1.88). Similarly, in the immobilized arm that received rTMS, there was a significant reduction in EMG activity (−31%, *p* < 0.05, BF_10_ = 2.30; Figure 5).

### The Effect of rTMS on Deconditioning of the Arm During Immobilization

**Figure 6:**
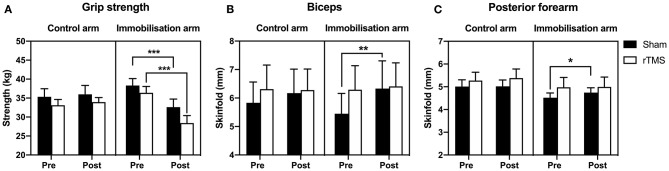
Effect of rTMS on immobilized-induced loss of grip strength and arm composition. **(A)** Arm immobilization induced a significant decrease in grip strength in both the Sham group (10% loss) and rTMS group (22% loss) (*p* < 0.0001). In the Sham group, there was an increase in biceps skinfold (**B**; *p* < 0.01) and posterior forearm skinfold (**C**; *p* < 0.05) of the immobilized arm. Such changes in arm composition were not observed in the rTMS group. *p < 0.05; **p < 0.01; ***p < 0.001.

To determine skeletal muscle decline, we measured maximal grip strength and changes in arm composition before and after the 72 h immobilization. A mixed-model ANOVA on grip strength with time and group as factors (as per EMG analysis) confirmed a significant effect of time: *F*_(1, 42)_ = 37.37, *p* < 0.0001, BF_10_ > 100; group: *F*_(3, 42)_ = 0.76, *p* > 0.05, BF_10_ < 1; time × group: *F*_(3, 42)_ = 20.49, *p* < 0.0001, BF_10_ > 1,000. Zero vs. seventy-two hour *t*-tests both *p* < 0.0001, BF_10_ > 100 in the immobilized arm with Sham and rTMS treatment. There was no significant loss of strength in the control arm in Sham or rTMS (*p* > 0.05, BF_10_ < 1, Figure 6A). However, in the immobilized arm, there was a significant loss of strength in Sham (*p* < 0.0001, BF_10_ > 150) that was not prevented by rTMS (*p* < 0.0001, BF_10_ > 1,000, Figure 6A). Arm volume was not significantly different after 72 h in the control or immobilized arm (or Sham/rTMS groups) when measured by plethysmography, mid-biceps, or mid-forearm circumference (*p* > 0.05, BF_10_ < 1, Table 2). Arm volume plethysmography mixed-model ANOVA: time: *F*_(1, 40)_ < 0.01, *p* > 0.05, BF_10_ < 1; group: *F*_(3, 42)_ = 0.41, *p* > 0.05, BF_10_ < 1; time × group: *F*_(3, 40)_ = 1.07, *p* > 0.05, BF_10_ < 1. Furthermore, in the control arm, there was no change in arm composition across the 72 h in Sham or rTMS groups when determined through skinfold measurements [*p* > 0.05, BF_10_ < 1: Table 2, biceps (Figure 6B) and posterior forearm (Figure 6C)]. However, in the immobilized arm in Sham, there was a significant increase in skinfold thickness of the biceps [Figure 6B; mixed-model ANOVA time: *F*_(1, 40)_ = 5.43, *p* < 0.05, BF_10_ = 2.51; group: *F*_(3, 40)_ = 0.08, *p* > 0.05, BF_10_ < 1; time × group: *F*_(3, 40)_ = 2.10, *p* = 0.12, BF_10_ < 1; +15%, *p* < 0.01, BF_10_ = 2.78 and *t*-test of 72 vs. 0 h *p* < 0.01] and posterior forearm [Figure 6C; mixed-model ANOVA time: *F*_(1, 41)_ = 5.83, *p* < 0.05, BF_10_ = 2.53; group: *F*_(3, 42)_ = 0.84, *p* > 0.05, BF_10_ < 1; time × group: *F*_(3, 41)_ = 1.62, *p* > 0.05, BF_10_ < 1. *t*-test of 72 vs. 0 h = *p* < 0.05, BF_10_ = 13.12. 7%, *p* < 0.05], which was seemingly prevented in the rTMS group.

**Table 2:** Effect of rTMS on arm composition with immobilization.

**Measure**	**Sham (*****n*** **=** **12)**				**rTMS (*****n*** **=** **12)**			
	**Control arm**		**Immobilization arm**		**Control arm**		**Immobilization arm**	
	**0 h**	**72 h**	**0 h**	**72 h**	**0 h**	**72 h**	**0 h**	**72 h**
**Arm volume**								
Arm volume (L)	2.09 ± 0.09	2.10 ± 0.09	2.20 ± 0.09	2.17 ± 0.08	2.11 ± 0.07	2.12 ± 0.07	2.20 ± 0.07	2.22 ± 0.08
Mid-biceps circumference (cm)	29.8 ± 0.9	29.1 ± 1.0	30.2 ± 0.9	30.2 ± 0.8	29.9 ± 1.0	29.8 ± 1.1	30.7 ± 0.9	30.6 ± 1.0
Mid-forearm circumference (cm)	26.2 ± 0.5	26.2 ± 0.6	26.5 ± 0.7	26.4 ± 0.7	26.9 ± 0.4	26.6 ± 0.5	26.8 ± 0.5	26.7 ± 0.5
**Arm composition**								
Biceps skinfold (mm)	5.8 ± 0.7	6.2 ± 0.9	5.5 ± 0.7	6.3 ± 1.0**	6.3 ± 0.8	6.3 ± 0.7	6.3 ± 0.8	6.4 ± 0.8
Triceps skinfold (mm)	10.3 ± 0.9	10.2 ± 0.9	11.7 ± 1.1	11.3 ± 1.1	10.2 ± 1.1	10.1 ± 1.1	9.4 ± 1.0	9.3 ± 1.0
Anterior forearm skinfold (mm)	4.9 ± 0.3	4.8 ± 0.3	4.4 ± 0.3	5.0 ± 0.3	4.9 ± 0.5	4.9 ± 0.5	5.0 ± 0.5	5.1 ± 0.5
Posterior forearm skinfold (mm)	5.0 ± 0.3	5.0 ± 0.3	4.5 ± 0.2	4.8 ± 0.2*	5.3 ± 0.4	5.4 ± 0.4	5.0 ± 0.4	5.0 ± 0.4

*Values denote mean ± SEM*.

* *p < 0.05 from 0 h;*.

** *p < 0.01 from 0 h*.

## Discussion

### Immobilization Induced a Significant Reduction in MEPs

The current study assessed whether immobilization affected motor excitability. Data from the immobilized arm indicated a reduction in excitability from 0 h (baseline) to 24 h, which became significantly different at 48 h. Notably, there was no significant difference in excitability between 24, 48, or 72 h. This indicates that the most severe reduction in motor excitability occurs in the first 48 h of immobilization. The reduction of cortical excitability reported here at 48 h post-immobilization is in line with findings from animal and human literature (Facchini et al., 2002; Rosenkranz et al., 2014). Indeed, reduced MEPs of the ring and little fingers have been shown following immobilization lasting from 8 to 72 h (Facchini et al., 2002; Langlet et al., 2012; Rosenkranz et al., 2014). Notably, in these studies only hand immobilization was used, whereas the present study utilized arm and hand immobilization, hence the methods are non-identical. A potential mechanism for this is modulation of the cortical elements responsible for generation of the late I-waves in the MEP (Facchini et al., 2002).

Data from the control arm did not show any significant changes in excitability. Therefore, it is unlikely that compensatory increases in corticospinal excitability in the non-immobilized motor pathway occurred during this time period. However, movement (physical activity) of the control (non-immobilized) arm was not quantified during the experiment. Hence, from these data we cannot know whether movement in the control arm reduced or increased during the 72 h, and how this relates to compensatory motor activity. Maximal grip strength and arm volume of the control arm, however, were unchanged from 0 to 72 h, which suggests that there were no significant changes in activity.

### rTMS Did Not Protect Against Loss of MEPs, Strength, or EMG Activity During Immobilization

Data indicated that at 0 h (baseline), the rTMS was effective at increasing motor excitability. However, the rTMS protocol did not appear to protect against loss of MEPs across the 72 h immobilization, nor did it protect against loss of strength, or EMG activity. Interestingly, whilst change has not been observed in the immobilized MEPs, data suggest that rTMS may have catalyzed unexpected changes in the motor pathway due to potential maintenance of arm composition. Indeed, there was an increase in biceps and posterior forearm skinfold thickness during immobilization in the Sham group that was not observed in the rTMS group. These changes in the Sham group were observed in context with no overall change in arm volume determined by both plethysmography, mid-biceps, and mid-forearm circumference. Whilst there are limitations of skinfold calipers for measurement of subcutaneous fat (Wells and Fewtrell, 2006), they show high test–retest reliability (intraclass correlation: 0.989) (Buxadé et al., 2018).

The observed increase in skinfold thickness in the biceps and posterior forearm could reflect an increase in subcutaneous fat, or, more likely, an increase in fluid during immobilization. Indeed, immobilization of a limb is capable of promoting both an increase in fat deposition (Manini et al., 2007) and an increase in fluid (edema) (Baz and Hassan, 2018). To the authors' knowledge, this is the first study to show that rTMS may modulate subcutaneous fat accumulation or edema during limb immobilization. In accordance with our findings, emerging evidence indicates that rTMS in animal models may have the potential to reduce edema (Cui et al., 2019). Further work is required to determine whether the effects of rTMS observed in the present study reflect protection against increases in fat content or the development of edema in the immobilized limb and the mechanisms which underpin such change.

## Limitations

The narrow age range and testing of only males may limit the generalizability of the results. Future studies should be replicated in females and in different age groups to increase the external validity of the findings. In this study, the plethysmography and skinfold measurements were unable to measure skeletal muscle and fat mass specifically and thus, there could have been a decrease in skeletal muscle mass and an increase in fat mass that is undetected by a change in arm volume. The specificity of the high frequency rTMS intervention may also limit the generalizability of the results. Whilst the results of this study do not indicate a protective effect of rTMS in our participant group, they cannot rule out rTMS as a possible rehabilitation technique in other contexts.

## Conclusion

Immobilization of the dominant arm induced a significant (and large, ~50%) reduction in MEPs within 48 h. Despite 20 Hz rTMS being effective in enhancing motor excitability at baseline, we did not find it protected against immobilization-induced loss of motor excitability, loss of EMG activity, or maximal grip strength when applied daily throughout immobilization. However, rTMS may have modulated factors such as fluid retention or fat accumulation, as there was no increase skinfold thickness at the biceps and posterior forearm following rTMS, but this was observed in the immobilized arm of the Sham group. Thus, loss of motor excitability appears to drive the loss of strength with immobilization, and 20 Hz rTMS could have some efficacy as a countermeasure against immobilization-induced changes in arm composition, but this requires further investigation and optimization.

## Data Availability Statement

The raw data supporting the conclusions of this article will be made available by the authors, without undue reservation.

## Ethics Statement

The studies involving human participants were reviewed and approved by Faculty of Science and Technology Research Ethics Committee. The patients/participants provided their written informed consent to participate in this study.

## Author Contributions

CG contributed to the conceptualization, data curation, formal analysis, funding acquisition, investigation, methodology, project administration, resources, supervision, validation, visualization, roles or writing—original draft, writing—review, and editing. AD, SJ, BO'H, AR, and K-AS contributed to the data curation, investigation, project administration, validation, writing—review, and editing. KS contributed to the investigation, supervision, roles or writing—original draft, writing—review, and editing. JB contributed to the resources, supervision, roles or writing—original draft, writing—review, and editing. HN contributed to the conceptualization, data curation, formal analysis, funding acquisition, investigation, methodology, project administration, resources, software, supervision, validation, visualization, roles or writing—original draft, writing—review, and editing. All authors contributed to the article and approved the submitted version.

## Conflict of Interest

The authors declare that the research was conducted in the absence of any commercial or financial relationships that could be construed as a potential conflict of interest.
